# Spontaneous regression of colloid cyst on the third ventricle: a case report with the review of the literature

**DOI:** 10.1186/s12883-022-02933-6

**Published:** 2022-10-29

**Authors:** Joo-Hwan Lee, Jong-Hwan Hong, Yeong Jin Kim, Kyung-Sub Moon

**Affiliations:** Department of Neurosurgery, Chonnam National University Hwasun Hospital and Medical School, 322 Seoyang-ro, Hwasun-eup, Jeollanam-do 58128 Hwasun-gun, South Korea

**Keywords:** Colloid cyst, Natural history, Spontaneous regression, Third ventricle

## Abstract

**Background:**

Colloid cyst (CC) is a rare and benign cyst found in the third ventricle near the foramen of Monro. Although the role of surgical resection is well established in symptomatic large-sized CC, it remains debatable whether surgical removal of CC with no symptoms or minimal symptoms is necessary.

**Case presentation:**

A 49-year-old male patient visited our institute for incidentally detected intracranial mass. MRI demonstrated typical, 12 mm-sized CC located in the third ventricle. It was noticed that the cyst spontaneously decreased in size from 12 mm to 4 mm on MRI at 18 months after the first visit.

**Conclusion:**

Although spontaneous regression is a very rare phenomenon in CC, regular imaging study and frequent neurologic examination can be an alternative option for well-selected, asymptomatic cases.

## Background

Colloid cyst (CC) is a benign cyst found in the third ventricle near the foramen of Monro [1]. It is rare, with an incidence of 0.5-1% of all intracranial tumors [2]. This lesion occurs rarely in children. Most cases of CC have been found in adults [3]. CC is especially common among people in their 30 and 50 s, with a possible familial predisposition. Its incidence rate decreases in age after adulthood.

Although CC is thought to be congenital, the pathogenesis of CC remains controversial [4]. This cyst shows a variety of clinical courses [5]. It can remain indolent for years and become discovered incidentally in asymptomatic patients. Clinically, this lesion presents in various ways, including headaches, gait disturbance, nausea, blurred vision and urinary incontinence. It is mainly associated with acute or chronic hydrocephalus. Sometimes CC can suddenly block foramen of Monro, causing drop attacks or sudden death [3, 6, 7]. Surgical resection is considered first for symptomatic CC treatment, even if hydrocephalus is absent [7]. In cases with minimal or no symptoms, the lesion could be surgically resected or observed through serial neuroimaging. Since spontaneous regression is very rare, we report such a case and discuss the pathological mechanism with a review of related literature.

## Case presentation

**Fig. 1:**
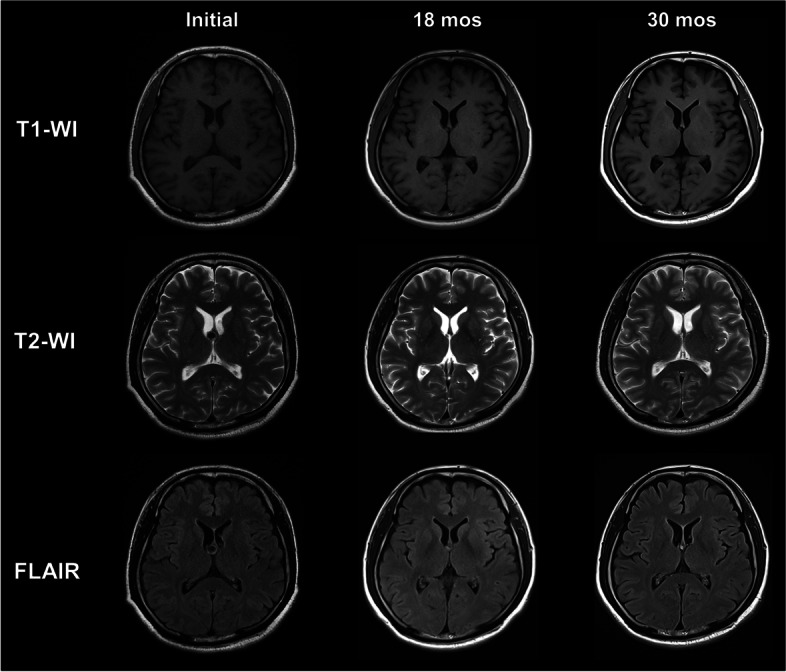
Chronologic evolution of the signal and morphology of a colloid cyst on serial MRI (T1- & T2-weighted, and FLAIR images). Follow-up MRI showed significant regression of the colloid cyst from 12 mm to 4 mm at 18 months after the initial diagnosis. This regression was maintained in follow-up MRI at 30 months

A 49-year-old male patient visited our institute for an incidentally detected intracranial mass. The patient had no underlying disease. He did not complain of any symptoms such as headaches or dizziness. On neurological and physical examination, there were no specific findings or neurological deficits associated with intracranial hypertension. Brain MRI revealed a 12 mm-sized CC located in the third ventricle. The cystic lesion was a round and well-demarcated lesion located in the right foramen of Monro and the upper third ventricle. The lesion was isointense on T1-weighted images and predominantly hyperintense on T2 with a hypointense focus within the lesion. The signal intensity pattern was the same on fluid-attenuated inversion recovery (FLAIR) images likely due to the highly proteinaceous nature of cyst fluid. There was neither hydrocephalus nor any signs of hemorrhage. The patient was followed up with regular imaging protocol. It was noticed that the cyst spontaneously decreased in size from 12 mm to 4 mm on MRI at 18 months after the first visit. As a result of follow-up at 30 months, the size of the lesion was maintained constant without any clinical symptoms (Fig. 1).

## Discussion and conclusions

CC is a pathologically benign intracranial mass [5]. The clinical course of the CC is not yet fully understood [6]. Although there are some well-known typical clinical patterns and radiologic findings, unpredictable neurological symptoms and variable radiological findings could be present [8]. Most CCs show indolent behavior. However, there are CC cases with sudden death [9] due to sudden blockage of cerebrospinal fluid (CSF) with brain herniation [5]. Based on this, even if there are no symptoms for large-sized CCs, surgical resection should be considered. However, whether surgical removal of CC with no symptom or minimal symptoms is necessary remains debatable.

The present case is the tenth report on spontaneous regression of CC [1, 7, 9–15] (Table 1). All previously reported cases were incidentally discovered or complained of minor symptoms. The initial size was reported to be 5 to 30 mm. Radiologically, the lesion decreased in size or disappeared over an observation period from 15 months to 9 years. There was no deterioration of symptoms during the observation period. Although the natural history of CC remains unclear, it was found that asymptomatic CC could be monitored through regular imaging based on a retrospective study of 162 CCs [6]. In the case of asymptomatic or incidentally detected CC, prophylactic surgery is also possible. However, the risk of complications that may occur during surgery should be considered. Moreover, physicians should remember that an asymptomatic CC naturally could decrease in size over time without specific treatment, as shown in reported cases including the present case.

**Table 1:** Summary of cases with spontaneous resolution of colloid cyst

Authors/year	Age	Sex	Symptoms	Symptom duration	Initial size	Duration to regression	Size at last follow-up	Follow-up duration	CCRS*
Motoyama et al.(2002) [7]	83	F	Gait disturbance, urinary incontinence, dementia	2 mos	NA	10 ds	small remnant	8 mos	0 + 1 + 1 + 0 + 0 = 2
Annamalai et al.(2008) [1]	35	M	Incidentally detected	-	5 mm	15 mos	disappeared	18 mos	1 + 0 + 0 + 0 + 0 = 1
Gbejuade et al.(2011) [9]	65	M	Headache, lassitude and forgetfulness	4 yrs	8 mm	19 mos	disappeared	19 mos	0 + 1 + 1 + 0 + 1 = 3
Peeters et al.(2016) [10]	46	F	Headache, altered mental status and amnesia	NA	2.852Cm^3^	5 mos	0.07Cm^3^	1 yrs	1 + 1 + 0 + 0 + 1 = 3
Turel et al.(2017) [11]	45	F	Headache, dizziness	6 mos	11 mm	2 yrs	disappeared	2 yrs	1 + 1 + 1 + 1 + 1 = 5
Mulcahy et al.(2020 [12]	51	M	Incidentally detected	-	18 mm	4 yrs	small remnant	9 yrs	1 + 1 + 1 + 0 + 0 = 3
Magalhães-Ribeiro et al.(2020) [13]	57	F	Intermittent scotoma	3 mos	10 mm	51 mos	disappeared	75 mos	1 + 1 + 1 + 0 + 0 = 3
Menéndez-Cortezón et al.(2020) [14]	2.5	F	Incidentally detected	-	30 mm	6 yrs	5 mm	60 mos	1 + 1 + 1 + 1 + 0 = 4
Cosgrove et al.(2020) [15]	67	M	Dizzness, left leg paresthesia, gait abnormalities	1 day	5 mm	4 yrs	3 mm	NA	0 + 0 + 0 + NA + 0 = 0 or 1
Our case	49	M	Incidentally detected	-	12 mm	18 mos	4 mm	30 mos	1 + 1 + 0 + 1 + 0 = 3

*F* Female, *M* Male, *ds* Days, *mos* Months, *yrs* Years, *NA* Not available

*CCRS; Colloid Cyst Risk Score (1 point for each variable; Age < 65, Diameter ≥ 7 mm, Anterior location, High signal intensity on FLAIR/T2-weighted image, Headache)

*CCRS 0,1,2; Low risk / CCRS 3 without hydrocephalus; Intermediate risk / CCRS 3 with hydrocephalus and CCRS 4,5; High risk

Although the pathophysiological cause of spontaneous regression has not been elucidated yet, a scenario in which the size decreases due to unrecognized cyst rupture is conceivable. Motoyama et al. were the first to report spontaneous regression of CC. They radiologically confirmed it as a cyst rupture. The cyst might rupture at an unknown time and all its contents could be absorbed into the ventricle, leaving only traces of the cyst wall in CC with no or minimal symptoms [7]. All spontaneous regression cases were up to 30 mm or less. The small volume of cyst wall suggests that it may appear lost on follow-up images. Aseptic meningitis might develop after rupture, although the probability is relatively low [1, 7, 9–16]. Further studies on changes in CC size are needed to understand the pathophysiological mechanisms of spontaneous regression.

For asymptomatic CCs, careful definition of the asymptomatic criteria is necessary because observation with follow-up imaging can be considered as a treatment option. Some patients say that they have no symptoms if the headache is minor, whereas others may report or feel it severe even if the headache is mild. In addition, if the size of CC increases, other signs of increased intracranial pressure should not be overlooked, and the patient should be educated.

Surgery should be considered, especially if symptoms develop during observation with an increase in the size of CC. One analysis has suggested predictors of asymptomatic to symptomatic progression, such as younger age, increased cyst size, ventricular dilatation, and increased signal on T2-weighted MRI [6]. Hussein et al. have proposed a Colloid Cyst Risk Score (CCRS) to determine the treatment of CC [16]. They suggested the following indicators as risk factors: under 65 years of age, at least 7 mm in length, anterior location, high signal intensity for FLAIR/T2 and lesion-related headaches. If CCRS is at least 3 points in total, asymptomatic cases are considered high risk. Of the 10 reported CC with spontaneous regression, 5 cases including our case were classified as medium-risk, 3 low-risk, and 2 high-risk (Table 1). Further studies are needed to predict worsening of CC based on the natural course of asymptomatic cases.

In conclusion, surgical resection is a main treatment for symptomatic or asymptomatic large CC. However, it sometimes regresses spontaneously without specific treatment. Therefore, well-selected asymptomatic CC can be observed with regular imaging study and frequent neurologic examination.

## Data Availability

The datasets generated during and/or analyzed during the current study are available from the corresponding author on reasonable request.

## Abbreviations

CC: Colloid cyst
MRI: Magnetic resonance image
FLAIR: fluid-attenuated inversion recovery
